# Mechanical unloading coupled with coronary reperfusion stimulates cardiomyocyte proliferation and prevents unloading-induced fibrosis after myocardial infarction

**DOI:** 10.1007/s00395-025-01147-4

**Published:** 2025-11-22

**Authors:** Sean O. Bello, Charanjit Singh, Filippo Perbellini, Prakash P. Punjabi, Cesare M. Terracciano

**Affiliations:** Imperial Centre for Translational and Experimental Medicine, Hammersmith Hospital, National Heart & Lung Institute, Imperial College London, London, W12 0NN UK

**Keywords:** Unloading, Infarction, Regeneration, Fibrosis, Reperfusion

## Abstract

Percutaneous left ventricular assist devices (LVADs) have become essential tools during coronary reperfusion in high-risk PCI. Significant reduction in infarct propagation is observed when mechanical unloading is coupled with reperfusion, but little is known of the effect this reduction in wall stress and extracellular matrix with LVADs has on the heart's regenerative capacity. This study investigates the effect coronary reperfusion coupled with mechanical unloading has on myocardial fibrosis, and the impact of these changes in extracellular matrix on the heart's regenerative potential. MI was induced by coronary artery ligation in Lewis rats. Hearts underwent permanent coronary ligation (AMI) or were reperfused after 90 min (AMI/R). In each group, hearts were either loaded (AMI-L or AMI/R-L) or unloaded (AMI-U or AMI/R-U). In the unloaded subgroup, the infarcted hearts were explanted after 90 min and transplanted into the abdomen of healthy recipients via heterotopic abdominal heart–lung transplantation. The recipient’s heart acted as control. Hearts were analysed on day 7. 30 hearts were studied. In the permanent ligation group, fibrosis increased in both the loaded and unloaded hearts with no significant rise in cardiomyocyte proliferation. After coronary reperfusion, no increase in fibrosis was observed with mechanical unloading but cardiomyocyte proliferation rose significantly (AMI/R-L vs AMI/R-U *p* = 0.0001). Cardiomyocyte proliferative rate in the loaded and unloaded hearts was 0.6% and 3.7%, respectively, after permanent ligation, and 0.5% and 10.4%, respectively, after coronary reperfusion. These data show that coronary reperfusion coupled with mechanical unloading reduces myocardial fibrosis and upregulates cardiomyocyte proliferation after myocardial infarction.

**Supplementary Information:**

The online version contains supplementary material available at 10.1007/s00395-025-01147-4.

## Introduction

Since the second half of the twentieth century, remarkable progress has been made in the treatment of cardiovascular diseases, in particular, coronary artery disease, with several major breakthroughs in the treatment for acute myocardial infarction (AMI) [1, 2]. However, these achievements have come at the expense of a vast increase in patients with congestive heart failure (CHF), a chronic consequence of AMI [3, 4]. In the United States currently, about 2 million patients with MI are living with CHF, with a 5-year survival rate of about 50%[5].

Thus, there remains a need to further optimise the management of AMI. The novel approach of coupling percutaneous left ventricular assist devices (LVADs) with coronary reperfusion during high-risk PCI has shown improved outcomes, with studies reporting significant reduction in infarct size with unloading [6–9].

This reduction in the propagation of post-infarct myocardial injury when reperfusion is coupled with mechanical unloading has been attributed to a reduction in myocardial oxygen consumption and the amelioration of ischaemia–reperfusion injury with LVAD use and LV unloading possibly due to increased collateral blood flow [10].

Ischaemia–reperfusion injury is a significant limiting factor in the current management of AMI [11–14]. Following an acute MI, early and successful reperfusion with the use of thrombolytic therapy or PCI limits infarct size and improves clinical outcome. However, despite these positive effects, reperfusion after an AMI has also been shown to be potentially harmful to the heart. Myocardial reperfusion injury involves a spectrum of detrimental effects on the injured heart that starts with the development of reversible functional impairment (myocardial stunning and reperfusion arrhythmias) to microvascular obstruction (no-reflow phenomenon) and ultimately, death of cardiac myocytes that were viable immediately before myocardial reperfusion [15, 16].

The pathophysiology of myocardial reperfusion injury has been extensively studied [12, 17]. It involves a reperfusion-induced exacerbation of the effects of acute cessation of blood flow to the heart seen in AMI [18]. During the ischaemic episode, there is an accumulation of lactic acid due to anaerobic respiration, and the loss of oxidative phosphorylation results in adenosine triphosphate (ATP) depletion with the consequent build-up of intracellular Na^+^ and Ca^2+^ ions and cellular oedema. Following reperfusion, a burst in reactive oxidant species leads to further depletion of ATP and mitochondrial damage. A further accumulation of intracellular Ca^2+^, leading to overload, cardiomyocyte hyper-contracture, and irreversible damage to membrane proteins and cytoskeleton, is also observed [17, 19]. Furthermore, reperfusion also triggers an inflammatory response, leading to endothelial injury, and microvascular obstruction, impaired perfusion, infarct size extension, and myocardial dysfunction.

Whilst decades of preclinical studies on measures to address myocardial reperfusion injury, such as ischaemic conditioning, pharmacological, and mitochondria-targeted cardioprotective therapies, have yielded promising results, translation to a clinical benefit for patients with reperfused AMI has been mostly disappointing [11, 20]. Thus, the reduction in infarct size propagation observed when mechanical unloading with LVADs is coupled with coronary reperfusion is promising [6, 21].

The potential for LVADs to facilitate myocardial recovery after myocardial injury is an area of much research interest [22–24]. It is well known that a consequence of mechanical unloading is a reduction in wall stress, that is, the tension in the myocardial fibres [25, 26]. By reducing myocardial wall stress, but also improving collateral circulation, cardiac excitation–contraction coupling, cardiac output and thus multi-organ function, and ameliorating the toxic neurohumoral milieu around the failing heart, LVADs are capable of stimulating significant improvement in myocardial function, a phenomenon described as reverse remodelling [21, 27, 28].

Reverse remodelling is associated with changes in the myocardium at multiple levels: whole organ, tissue, cellular, and molecular. The “main players” in this process include the cardiomyocytes, the extracellular matrix (ECM), and the microvascular system, as well as neuroendocrine mechanisms. The impact of cardiomyocyte regeneration in reverse remodelling is yet to be explored but might play a key role in the myocardial recovery observed with LVADs [29].

A well-held paradigm up until the early parts of this century was that the heart is a post-mitotic organ and all cardiac myocytes are terminally differentiated and thus incapable of re-entering the cell cycle. More recently, however, a few remarkable studies have provided evidence to the contrary [30, 31]. Various research groups now show that the adult mammalian heart, by itself, possesses an intrinsic form of cellular homeostasis that permits regeneration and formation of new cardiac myocytes and vasculature [2, 32–35]. To date, however, the impact of haemodynamic unloading after MI on the heart’s regenerative capacity remains unclear. Thus, the effect of unloading coupled with coronary reperfusion after MI on load-sensitive cell-intrinsic signalling pathways that regulate cardiomyocyte proliferation, such as the Hippo pathway via the expression of phosphorylated YAP (pYAP), is investigated in this study [36–38].

Finally, a few contemporary studies have identified that the cardiac ECM has a significant influence on the degree of reverse remodelling that occurs during mechanical unloading with LVADs [39, 40]. Myocardial fibrosis increases after unloading [41–43]; however, how this occurs is not well understood. It is thought to be related to inhibitory effects of unloading on the autoregulated coronary collateral microcirculation resulting in propagation of the ischaemic insult [16, 44]. This highly autoregulated microcirculation is known to help match myocardial blood flow to changes in myocardial oxygen consumption (MV0_2_) following occlusion of a large coronary vessel [16, 21]. Thus, we hypothesise that by coupling mechanical unloading with return of blood flow through the ligated major coronary vessel, these effects on microvasculature and fibrosis will be counteracted [45, 46].

In this study, using rodent models of AMI, coronary reperfusion, and mechanical unloading via heterotopic abdominal heart and lung transplantation (HAHLT), we investigated the effect of mechanical unloading, coupled with coronary reperfusion, on the progression of myocardial fibrosis and the cardiomyocyte proliferative capacity of the infarcted heart.

## Materials and methods

### AMI, coronary reperfusion, and HAHLT rodent models

Personal, project, and establishment animal licences were granted by the UK Home Office as required by the UK Animals Scientific Procedures Act (ASPA) 1986. All animal procedures were performed in accordance with the National Institute for Health (NIH) Guide for the Care and Use of Laboratory Animals (National Research Council 2011). In brief, syngeneic male Lewis rats were anaesthetised using the inhalation anaesthetic agent isoflurane at 3–5% for induction and 1.5–2.5% for maintenance. The anaesthetic circuit used was the Bain’s co-axial facemask. Oxygen flow was kept at 3L/min for induction and 1.5–2 L/min for maintenance. Animals were then intubated using a 16 G vascular cannula and general anaesthesia achieved via a volume controlled rodent ventilator (Model 683 Rodent ventilator, Harvard 101Apparatus Ltd, UK). The tidal volume was kept at about 6-8 ml/kg with a ventilatory rate of about 40 breaths/min and body temperature of 37 °C. Intraoperative pain relief consisted of a single subcutaneous dose of buprenorphine at 0.12 mg/kg. Adequate hydration was achieved by giving 1 ml bolus of warm saline subcutaneously every hour.

### Permanent coronary artery ligation and ischaemia–reperfusion

Once adequacy of anaesthesia was confirmed by loss of pedal reflexes, the animal’s chest wall was shaved, prepped, and draped, a left anterolateral thoracotomy was made over the fourth rib, and the proximal left anterior descending artery (LAD) was ligated using 6/0 prolene sutures.

After coronary artery ligation, visual assessment of the apex of the heart was carried out to determine onset of the infarct. The lungs were inflated and the chest wall temporarily closed. Animals were then left on the ventilator for 90 min. This was taken as sufficient time to allow transmural myocardial infarction to occur.

In the loaded permanently ligated subgroup (AMI), the animal was recovered after 90 min. In the loaded reperfusion subgroup (AMI/R-L), the chest wall was reopened after 90 min and the ligature to the proximal LAD released to return blood flow to the infarct territory. The chest wall was then closed and the animal recovered [20]. In the unloading subgroup (AMI/U or AMI/R-U), after 90 min the torso was prepped with povidone iodine and draped. The abdominal aorta was then accessed via a midline laparotomy and adequate heparinisation was achieved by injecting heparin at 10,000 IU/Kg into the inferior vena cava (IVC) using a 30G needle. The donor abdominal aorta was cannulated with a 20-gauge arterial line (Leadercath arterial, Vygon UK Ltd, FSQ049) using a catheter-over-wire technique and this was used to inject 50 ml of cold St Thomas II cardioplegia solution into the donor circulation, thus arresting the donor heart (permanently ligated or AMI/R) in diastole. The IVC was simultaneously transected to prevent overload, and euthanasia was achieved via exsanguination. The sternum was cut open and the heart explanted after ligating the IVC and superior vena cava (SVC) using 4/0 Mersilk sutures. The heart was then implanted into the abdomen of a syngeneic recipient via HAHLT.

### HAHLT

HAHLT technique was used as the model of mechanical unloading [25].

HAHLT in rats is a well-established technique that had been initially employed to facilitate studies in cardiac transplant immunology but has also been optimised to study the effects of mechanical unloading on the failing heart [25, 47–49]. It involves anastomosing the heart and lung *en bloc* from a donor onto the abdominal aorta of a healthy recipient. This model allows a much less profound or partial unloading of the LV which is a more physiological representation of LVAD-induced mechanical unloading [50]. Only a single anastomosis of the ascending aorta of the donor heart to the abdominal aorta of the recipient is completed. As the donor IVC and SVC are ligated and the pulmonary circulation remains intact, the LV receives additional blood from the right ventricle (RV), via the pulmonary veins after passing through the lungs (illustrated in Figure S1, supplementary page 4).

**Fig. 1:**
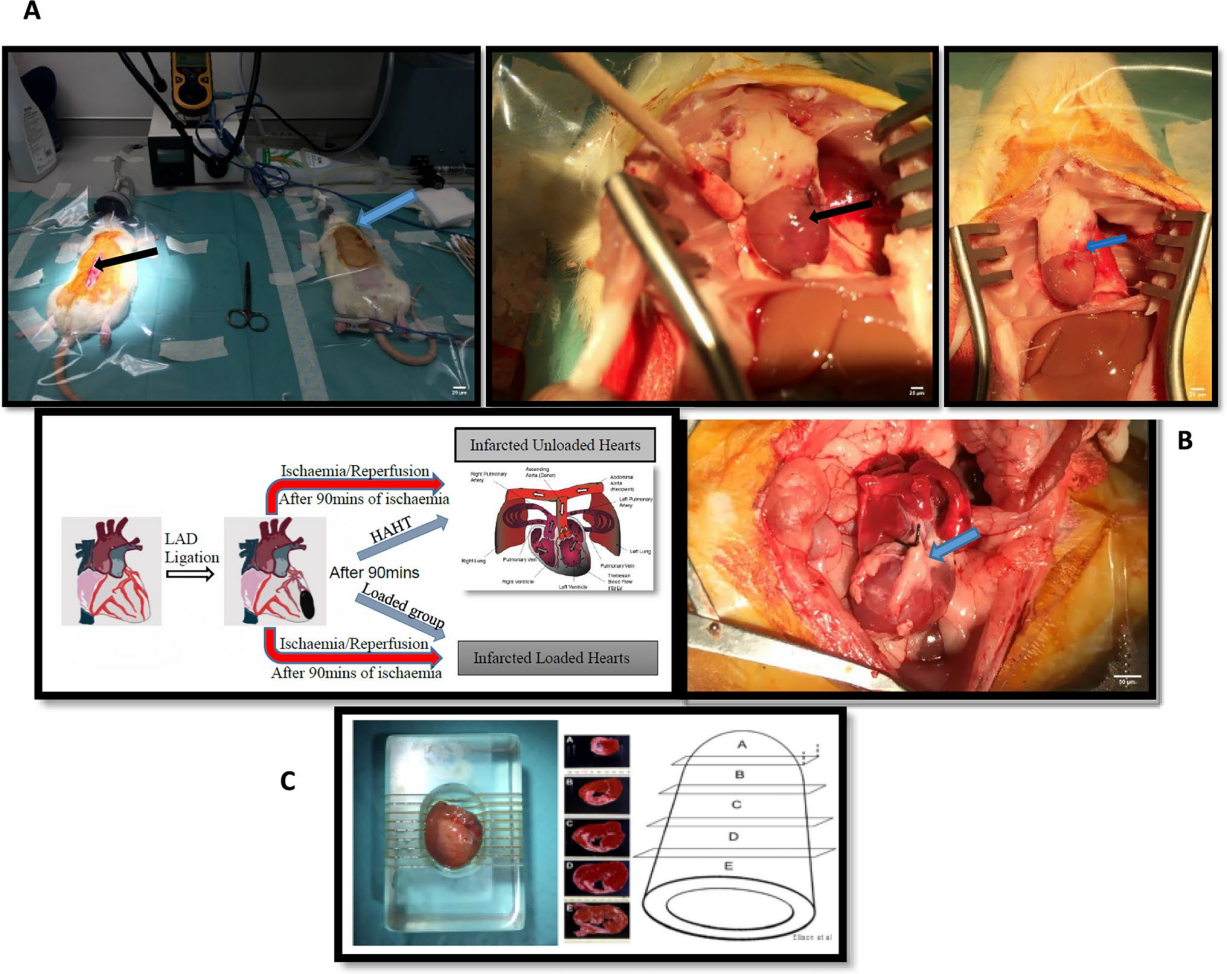
Representative images summarising the procedural steps involved in animal model creation. (A) After 90 min of MI, hearts were either left with permanent coronary artery ligation or reperfused by ligature release. Rats were then either recovered (loaded group) or hearts were explanted and implanted into the abdomen of a healthy recipient for mechanical unloading via HAHLT. Note the blue arrow in the left image in **A** pointing at the closed left thoracotomy wound following coronary ligature. The rat on the left is being prepared for HAHLT with the black arrow pointing at the laparotomy incision. The middle image in **A** shows the ligated heart after undergoing diastolic arrest following infusion of cold crystalloid cardioplegia solution prior to explantation for HAHLT, note the black arrow pointing at the unblanched infarcted territory downstream from the LAD ligature (blue arrow). The right image in **A** shows the previously well demarcated infarct territory has now been blanched (blue arrow) following ligature release and infusion of crystalloid cardioplegia, indicating adequate coronary reperfusion. Left image in **B** is a schematic representation of the steps described in **A** and includes a graphic illustrating HAHLT. In HAHLT, the ascending aorta of the donor heart is anastomosed to the abdominal aorta of the recipient and as the SVC and IVC are ligated, only the coronary flow down the donor’s aorta returns to the LV. The right image in **B** shows the donor heart in the abdomen of the recipient, note the blue arrow pointing at the IVC ligature. Following heart harvest, the atria were excised and the ventricles placed in an acrylic zivic rat heart slicer matrix **C** where five 2 mm coronal section slice intervals **A-E** were obtained for analysis. Right-sided image of **C** reproduced with permission from Ellison et al. 2011. [33]

The surgical steps have been previously described. In brief, both the recipients’ and donor torsos were shaved, prepped with povidone iodine, and draped as shown in Fig. 1. The recipients’ abdominal aorta was accessed via a midline laparotomy incision, and with the aid of a microscope, a 0.5 cm midline longitudinal aortotomy was carried out and prepared for anastomosis after achieving proximal and distal vascular control. The donor abdominal aorta was also accessed and the donor heart explanted as described above.

### Animal recovery and post-operative care

Recovered animals were observed in the operating theatre for an hour following weaning from general anaesthesia at which point signs of pain or distress were acted upon in accordance with Home Office regulations. Post-operative analgesia was achieved by injecting carprofen subcutaneously at 0.5 mg/kg at the end of the operation and once daily for two days. Antibiotic cover consisted of enrofloxacin given subcutaneously at 1 mg/kg at the end of the operation and daily for two days. Animals were checked twice a day for the first 3 post-operative days. Daily weights and health checks were performed thereafter.

30 male syngeneic Lewis rats weighing 250–300 g (Charles River, UK) were utilised to generate models of AMI, coronary reperfusion (R), and mechanical unloading (U) via HAHLT.

Detailed description of the operative procedures including heart harvest on day 7, tissue preparation, and experimental techniques are provided in the supplementary material.

### Experimental groups

As described above, MI was induced by either permanently ligating the LAD (AMI) or reperfusing the vessel after 90 min (AMI/R). In each group hearts were either loaded (AMI-L or AMI/R-L) or unloaded (AMI-U or AMI/R-U). The animals of the loaded subgroup were recovered, whilst in the unloaded subgroup, the infarcted hearts were explanted after 90 min and transplanted into the abdomen of healthy recipients via HAHLT. The recipient’s heart acted as control and was depicted by green bars in the results.

Hearts were explanted on day 7 for histology and immunohistochemistry (Fig. 1).

4 subgroups were formed: AMI loaded (AMI-L), AMI reperfusion loaded (AMI/R-L), AMI unloaded (AMI-U), and AMI reperfusion unloaded (AMI/R-U).

Detailed annotations for each group are provided in the supplementary material. All animals were randomly assigned identification codes, and these were blinded to the primary investigator who carried out all the experiments as detailed in the supplementary material.

Histological analysis of frozen tissue sections for the quantification of myocardial fibrosis.

15 μm cryosections were prepared and processed with histological stains to assess the degree of fibrosis in the rat models of MI. Sirius red/Fast green collagen staining was employed using a standardised protocol. Images of the stained sections were obtained with widefield microscopy and with the aid of imageJ thresholding tool, areas of collagen deposition were contrasted from areas of healthy myocardium in the entire left ventricle of all the hearts in each subgroup (Fig. 3), and the extent of myocardial fibrosis was quantified (illustrated in supplementary figure S6A & S6B, supplementary methods).

### Mode of detection of proliferating cells

Immunohistochemistry of frozen cross sections using the proliferative marker Ki67 was employed to determine cell proliferation. Ki67’s sole expression during the active phases of the cell cycle (G1, S, G2, and mitosis) distinguishes it from markers that are heavily influenced by DNA damage and repair such as the proliferating cell nuclear antigen or the thymidine analogue Bromodeoxyuridine (BrdU) [51, 52].

### Western blotting

Western blotting technique was used to determine the expression profiles of αSMA and pYAP.

Details of the above methods are provided in the supplementary methods.

### Statistical analysis

The degree of myocardial fibrosis and cardiomyocyte proliferation was compared between the loaded and unloaded permanently ligated––and ischaemia-reperfused––hearts. A two-tailed *t*-test was used for the comparison between independent groups of normally distributed data as determined by Kolmogorov–Smirnov testing. One-way analysis of variance (ANOVA) followed by Bonferroni post hoc test for individual significant differences was used. All statistical analyses were done using Prism 8 software (GraphPad Software, Inc.). Five animals (*n* = 5) were studied in each subgroup. The results are presented as means ± SD. The significance threshold was set at *P* < 0.05 for all data. *Denotes P < 0.05, **denotes *P* < 0.01, and ***denotes *P* < 0.001.

## Results

### Effect of mechanical unloading on myocardial fibrosis after acute myocardial infarction

**Fig. 2:**
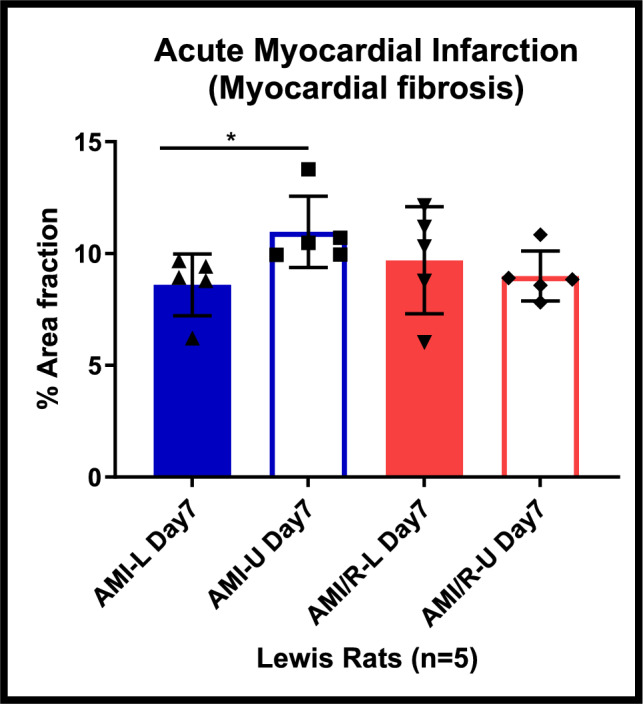
Differences in myocardial interstitial fibrosis between loaded and unloaded hearts after permanent coronary artery ligation and coronary reperfusion. Images of the stained sections were obtained with widefield microscopy and with the aid of imageJ thresholding tool, areas of collagen deposition were contrasted from areas of healthy myocardium in the entire left ventricle of all the hearts in each subgroup, and the extent of myocardial interstitial fibrosis was quantified. Note the significant increase in fibrosis noted after mechanical unloading in the permanently ligated hearts was not observed in the mechanically unloaded reperfused hearts. Blue bars represent permanently ligated hearts, whilst red bars are the reperfused hearts. The full bars are the loaded hearts, whilst the empty bars represent the mechanically unloaded hearts. There were 5 rat hearts studied per group. One-way analysis of variance (ANOVA) followed by Bonferroni post hoc test for individual significant differences was used. All statistical analyses were done using Prism 8 software (GraphPad Software, Inc.). The results are presented as means ± SD. The significance threshold was set at *p* < 0.05 for all data. *denotes *P* < 0.05, **denotes *P* < 0.01, and ***denotes *P* < 0.001

**Fig. 3:**
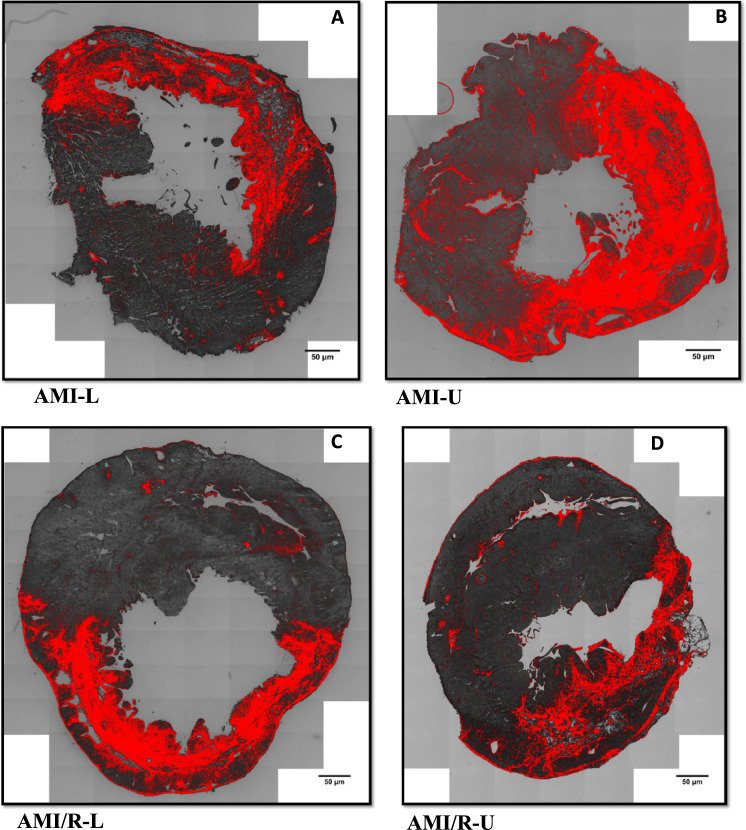
Histological samples at day 7. **A** Myocardial fibrosis in AMI-L with increase in fibrosis evident after mechanical unloading in the AMI-U **B**. In the coronary reperfusion group **C, D,** however, mechanical unloading in the AMI/R-U **D** did not significantly change the extent of fibrosis compared to that in the loaded AMI/R-L heart **C**. Areas of fibrosis was determined by Sirius red staining and quantified using ImageJ software

After AMI, mechanical unloading in the permanently ligated hearts was associated with a statistically significant increase in interstitial fibrosis compared to loaded hearts at day 7 (AMI-L vs AMI-U *P* = 0.0363). Figure 2 shows that, in the coronary reperfusion group, mechanical unloading after acute MI was not associated with increase in fibrosis (AMI/R-L vs AMI/R-U *P* = 0.5711) as illustrated in Figs. 2 and 3.

In the unloaded reperfused hearts, areas furthest from the infarct site showed less fibrosis (Fig. 3D) compared to that observed in the unloaded permanently ligated hearts (Fig. 3B).

### Regional evaluation of cardiomyocyte proliferation in permanently ligated and coronary reperfused hearts

**Fig. 4:**
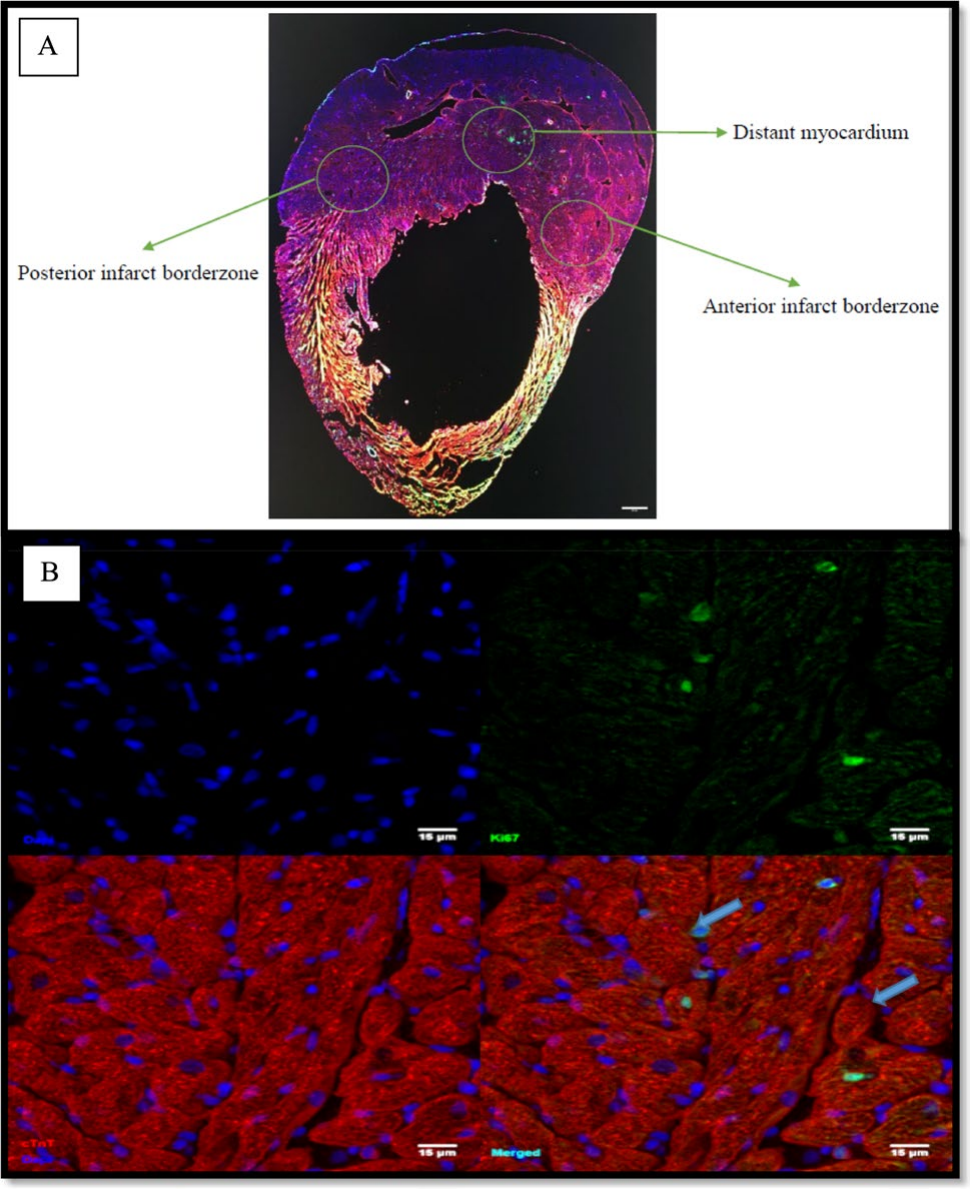
Bright-field microscopy **A** of a 15 μm frozen cross section of a Lewis rat heart after immunohistochemistry staining using cTnT (red) to identify cardiomyocytes, DAPI (blue) for the nuclei, and Ki67 (green) to detect proliferating cells. The three regions evaluated to determine cardiomyocyte proliferative response to MI are shown. **B** Confocal microscopic image of the tissue cross section showing 2 proliferating cardiomyocytes (blue arrows)

Analysis of cardiomyocyte proliferation was completed at day 7 as several studies have reported significant cardiomyocyte regenerative responses to myocardial injury within this time point [53–55]. Regional analysis of proliferation was completed to facilitate the determination of the differential effects of mechanical unloading on cardiomyocyte proliferation after MI in areas of the myocardium close to the primary infarct compared to more distant myocardium. The regions assessed are illustrated in Fig. 4A.

The anterior infarct borderzone represents the area between the anterior wall of the right ventricle, septum, and the anterior aspect of the LV free wall. The posterior infarct borderzone is the area between the posterior wall of the right ventricle, septum, and the posterior aspect of the left ventricular free wall. Finally, the distant myocardium is the midpoint of the interventricular septum. Cardiac troponin (cTnT) positive cells (stained red in Fig. 4B) expressing Ki67 (green) in their nuclei (blue) were counted as proliferating cardiomyocytes.

#### Anterior infarct borderzone

**Fig. 5:**
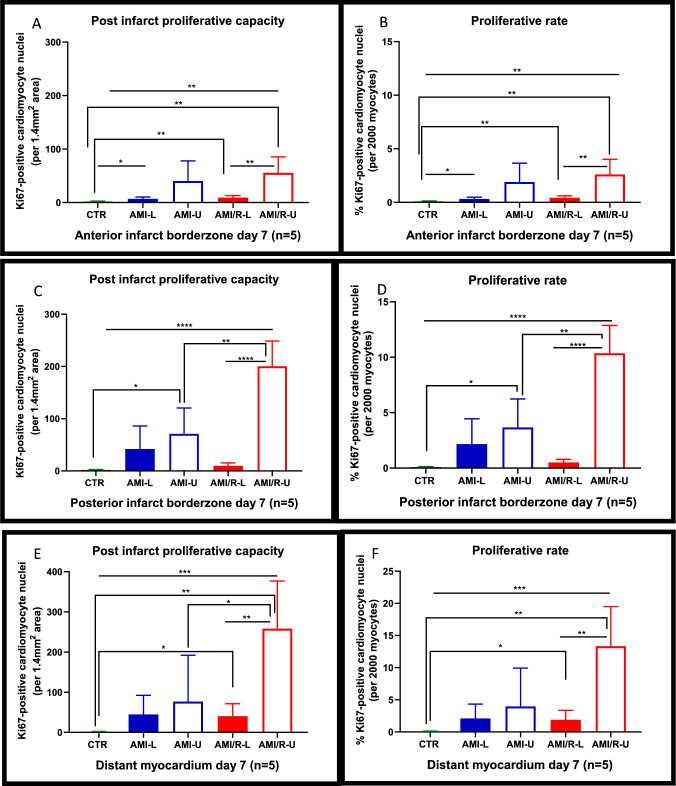
Regional analysis of proliferation was completed to facilitate the determination of the differential effects of mechanical unloading on cardiomyocyte proliferation after MI in areas of the myocardium close to the primary infarct compared to more distant myocardium. Three regions per rat were chosen each measuring 1.4 mm^2^ (anterior infarct borderzone, posterior infarct borderzone, and distant myocardium), from where proliferating cardiomyocytes were counted. **A-F** Regional proliferation of Ki67-positive cardiomyocytes in the anterior and posterior infarct borderzone, and distant myocardium 7 days after permanent coronary artery ligation or coronary reperfusion. In each illustrative figure, the filled bars represent loaded hearts and the empty bars unloaded hearts. The permanently ligated coronary artery group are in blue and the coronary reperfusion group in red. The proliferative capacity reflects the total number of Ki67-positive cardiomyocyte in the chosen region of myocardium. The proliferative rate is the number of proliferating cardiomyocytes per 2000 cardiomyocytes in each chosen region. One-way analysis of variance (ANOVA) followed by Bonferroni post hoc test for individual significant differences was used. All statistical analyses were done using Prism 8 software (GraphPad Software, Inc.). The results are presented as means ± SD. The significance threshold was set at *p* < 0.05 for all data. * denotes *P* < 0.05, **denotes *P* < 0.01, and ***denotes *P* < 0.001

In the anterior infarct borderzone at day 7, cardiomyocyte proliferation was 4 times higher in the loaded infarcted hearts compared to control after permanent coronary artery ligation (AMI-L vs CTR *p* = 0.0185). After mechanical unloading, there was no statistically significant difference in cardiomyocyte proliferation between the loaded and unloaded hearts in the anterior infarct borderzone after permanent coronary artery ligation (Fig. 5A).

After coronary reperfusion, there was a statistically significant 5 times increase in cardiomyocyte proliferation in the loaded hearts compared with control (AMI/R-L vs CTR *p* = 0.0027). Mechanical unloading coupled with coronary reperfusion stimulated a significant increase in cardiomyocyte proliferation compared to both the control hearts (AMI/R-U vs CTR *p* = 0.004) and the loaded reperfused hearts (AMI/R-U vs AMI/R-L *p* = 0.0092) as shown in Fig. 5A. The proliferative rate in the control, loaded, and unloaded hearts was 0.1%, 0.3%, and 2% in the permanently ligated hearts, and 0.1%, 0.4%, and 2.6% in the reperfused hearts, respectively (Fig. 5B).

#### Posterior infarct borderzone

In the posterior infarct borderzone at day 7, permanent coronary artery ligation was associated with an increase in cardiomyocyte proliferation in the loaded hearts compared to control, but this was not statistically significant (AMI-L vs CTR *p* = 0.0781) (Fig. 5C).

There was also no statistically significant difference in cardiomyocyte proliferation between the loaded and unloaded permanently ligated hearts (Fig. 5C). However, in the coronary reperfusion group, there was a statistically significant 5 times increase in cardiomyocyte proliferation in the loaded hearts compared to control (AMI/R-L vs CTR *p* = 0.0118). After mechanical unloading coupled with coronary reperfusion, cardiomyocyte proliferation rose quite significantly compared to the loaded reperfused hearts (AMI/R-U vs AMI/R-L *p* = 0.0001). (Fig. 5C). Mechanical unloading coupled with coronary reperfusion was also associated with a significant increase in proliferation when compared to unloading alone without reperfusion (AMI/R-U vs AMI-U *p* = 0.0031). The proliferative rate in the control, loaded, and unloaded hearts was 0.1%, 0.6%, and 3.7% in the permanently ligated hearts, and 0.1%, 0.5%, and 10.4% in the reperfused hearts, respectively (Fig. 5D).

#### Distant myocardium

In the distant myocardium at day 7, there was also no statistically significant difference in cardiomyocyte proliferation between the loaded and unloaded hearts after permanent coronary artery ligation (Fig. 5E). However, after coronary reperfusion, there was a statistically significant increase in cardiomyocyte proliferation in the loaded hearts compared to control (AMI/R-L vs CTR *p* = 0.0248) and this increase was even larger after mechanical unloading (AMI/R-U vs CTR *p* = 0.0013). Mechanical unloading coupled with coronary reperfusion resulted in a large and statistically significant increase in cardiomyocyte proliferation compared to the loaded reperfused hearts (AMI/R-U vs AMI/R-L *p* = 0.0042). In addition, there was a statistically significant increase in proliferation in the unloaded reperfused hearts compared to the unloaded permanently ligated hearts (AMI/R-U vs AMI-U p = 0.0402) (Fig. 5E).

The proliferative rate in the control, loaded, and unloaded hearts was 0.1%, 2.1%, and 3.9% in the permanently ligated hearts, and 0.1%, 1.9%, and 13.4% in the reperfused hearts, respectively (Fig. 5F).

### Effect of Mechanical Unloading on the Dynamic Expression Profiles of αSMA and pYAP after Myocardial Infarction

#### Effect of mechanical unloading on αSMA expression after acute myocardial infarction

**Fig. 6:**
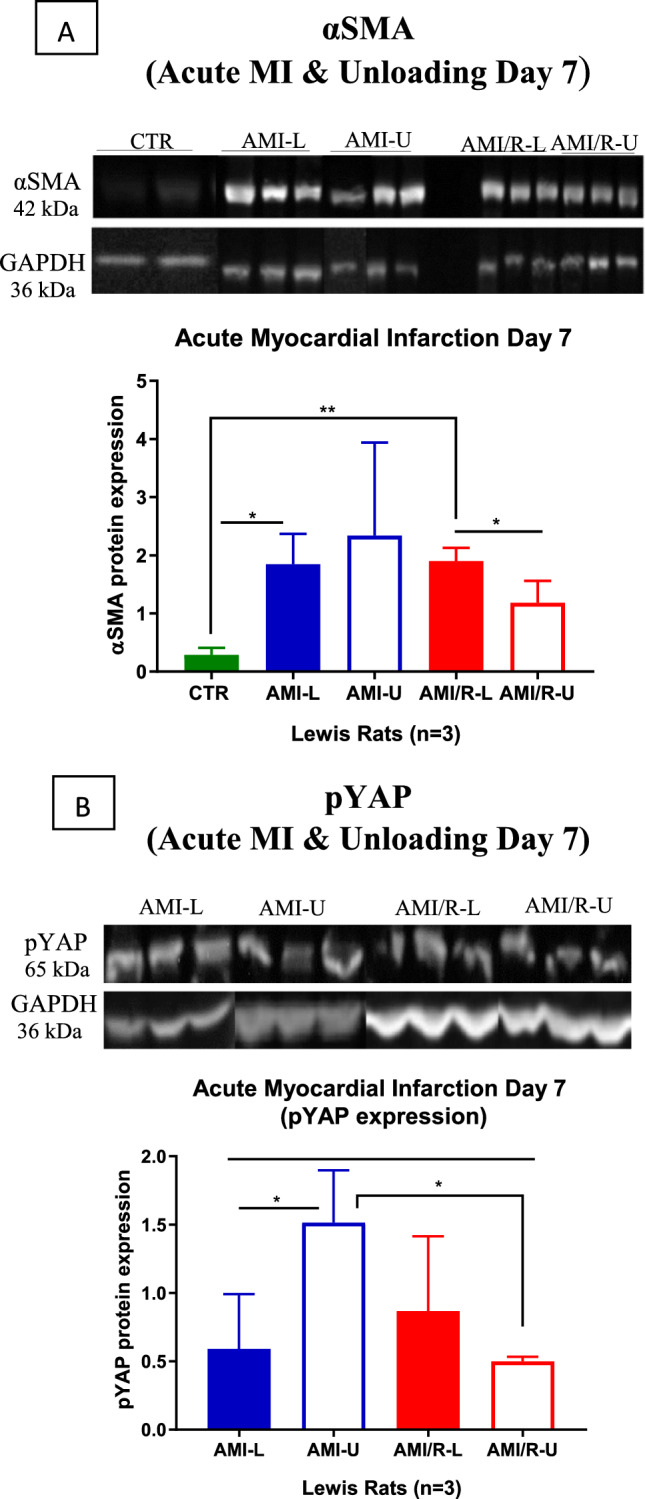
**A** and **B** showing the effects of mechanical unloading on the expression profiles of αSMA and pYAP after 7 days of permanent coronary artery ligation and coronary reperfusion. Three rats were tested in each group. In each illustrative figure, the filled bars represent loaded hearts and the empty bars unloaded hearts. The permanently ligated coronary artery group are in blue and the coronary reperfusion group in red. One-way analysis of variance (ANOVA) followed by Bonferroni post hoc test for individual significant differences was used. All statistical analyses were done using Prism 8 software (GraphPad Software, Inc.). The results are presented as means ± SD. The significance threshold was set at *p* < 0.05 for all data. *denotes *P* < 0.05, **denotes *P* < 0.01, and ***denotes *P* < 0.001

In the loaded permanently ligated heart, AMI resulted in a statistically significant rise in αSMA protein expression compared to control at day 7 (AMI-L vs CTR *p* = 0.0291) as shown in Fig. 6A. Mechanical unloading was associated with a further rise in αSMA protein expression, but this was not statistically significant. In the coronary reperfusion group, αSMA protein expression also rose significantly in the loaded hearts compared to control (AMI/R-L vs CTR *p* = 0.0020). However, in contrast to the permanent ligation group, mechanical unloading coupled with coronary reperfusion led to a statistically significant decrease in αSMA protein expression (AMI/R-L vs AMI/R-U *p* = 0.0483) (Fig. 6A).

#### Effect of mechanical unloading on pYAP expression after acute MI

At day 7 after permanent coronary artery ligation, there was a statistically significant increase in pYAP expression in the unloaded hearts compared to the loaded hearts (AMI-U vs AMI-L *P* = 0.0450) (Fig. 6B).

**Fig. 7:**
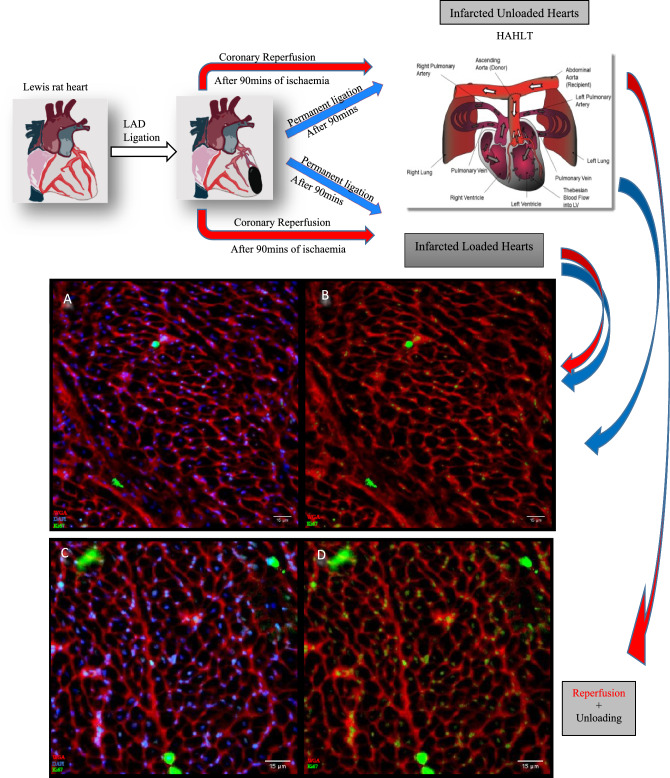
Schematic diagram showing infarcted Lewis rat hearts after either permanent LAD ligation (blue arrows) or coronary reperfusion (red arrows). Both groups were then either left loaded or the hearts explanted and mechanically unloaded via heterotopic abdominal heart and lung transplantation (HAHLT). **A-D** are immunohistochemistry stains for the quantification of cardiomyocyte proliferation. Red is the membrane marker wheat germ agglutinin (WGA). Blue is the nuclei marker (DAPI). Green is the proliferative marker (Ki67). **B** is the same image as **A**, but with the blue nuclei stain silenced to allow better visualisation of Ki67 stain. Similarly **D** is the same image as **C** but with the nuclei stain silenced. Cardiomyocyte proliferative capacity was stunted in the loaded hearts irrespective of ligated vessel reperfusion **A-B**. Only when mechanical unloading was coupled with coronary reperfusion did significant augmentation of cardiomyocyte proliferation occur **C-D**. Embedded HAHLT figure reproduced with permission from Ibrahim 2013 [25]

However, mechanical unloading coupled with coronary reperfusion was associated with a statistically significant decrease in pYAP protein expression compared to the unloaded permanently ligated hearts (AMI/R-U vs AMI-U *p* = 0.0104) (Figs. 6B and 7).

## Discussion

This study shows that mechanical unloading coupled with coronary reperfusion is associated with an increase in the heart’s endogenous cardiomyocyte proliferative capacity. Increases in cardiomyocyte proliferative rate after myocardial injury ranged from 0.3% to 2.1% in the loaded permanently ligated and reperfused hearts. However, after mechanical unloading was coupled with coronary reperfusion, there was a much larger and statistically significant rise in proliferation of new cardiomyocytes, particularly in the regions of the myocardium furthest away from the primary area of infarct, i.e. 2.6%, 10.4%, and 13.4%, in the anterior infarct borderzone, posterior infarct borderzone, and distant myocardium, respectively.

This finding supports reports from studies in several animal models that have shown that cardiomyocytes at the periphery of MI undergo endomitosis and polyploidisation instead of conventional mitosis with many of these cardiomyocytes harbouring a polyploid as opposed to the normal diploid genome [26]. Upon relief of wall stress in these hearts via mechanical unloading, there was a more than twofold increase in diploid cardiomyocytes with a significant decrease in the number of polyploid cardiomyocytes. It appears that the vast polyploidy of cardiomyocytes in the injured heart is a result of hypertrophic stimuli being associated with repeated rounds of DNA synthesis with an incomplete attempt to enter mitosis that has been blocked after completion of DNA replication (endomitosis). When haemodynamic stress is relieved by unloading and the hypertrophic stimulation is decreased, this blockade is also released, resulting in karyokinesis and the formation of binucleated cardiomyocytes, cytokinesis, and real cell duplication [26]. Hence, the favourable conditions during LVAD support, which reduces wall stress and improves the neurohumoral environment, promote conditions for cardiac regeneration [56].

To further assess the impact mechanical unloading coupled with coronary reperfusion on the heart’s endogenous cardiomyocyte proliferative capacity, the effect of unloading on the activity of one of the cell-intrinsic signalling pathways that regulate the proliferation of new cardiomyocytes was investigated. The expression of YAP and pYAP impacts cardiomyocyte proliferation [38, 46]. YAP’s presence in the cell nucleus is vital for cellular proliferation. When phosphorylated, YAP becomes extruded from the nucleus and is in the cytosol as pYAP with consequent reduction in cell proliferation. Thus, high levels of pYAP are associated with a downregulation of cellular proliferation [38, 46, 57]. This study showed that mechanical unloading coupled with coronary reperfusion resulted in a statistically significant decrease in the levels of pYAP compared to the unloaded permanently ligated hearts. This correlates well with the high levels of cardiomyocyte proliferation seen on immunohistochemistry when mechanical unloading is coupled with coronary reperfusion. In the absence of coronary reperfusion, mechanical unloading alone was associated with a statistically significant increase in the expression of pYAP corresponding with the muted regenerative response observed in this setting.

The augmented endogenous cardiomyocyte proliferative capacity observed in this study occurred in areas with decreased myocardial interstitial fibrosis. It is worth noting that myocardial fibrosis increased when the permanently ligated hearts were mechanically unloaded. There was a significant rise in fibrosis after 7 days (21%) of unloading with no significant increase in cardiomyocyte proliferation. This observed increase in fibrosis with mechanical unloading has been previously reported [43]. Remarkably, however, when mechanical unloading was coupled with coronary reperfusion, there was no significant increase in myocardial fibrosis and cardiomyocyte proliferation rose significantly. This supports our hypothesis that coupling mechanical unloading with coronary reperfusion ameliorates the deleterious effects of myocardial reperfusion injury after AMI.

Understanding the underlying mechanism by which increase in myocardial fibrosis occurs in the unloaded permanently ligated hearts and not in the unloaded reperfused hearts is in itself of major clinical significance. We have not established a mechanism here, but the findings from this study suggests a link between the re-establishment of blood flow in a large less autoregulated vessel and the amelioration of ongoing ischaemic injury to the heart during mechanical unloading. This implies a possible cardioprotective effect of coupling mechanical unloading with coronary reperfusion on the harmful pathophysiological processes of ischaemia–reperfusion injury. Furthermore, as the main difference between the two groups studied (permanent LAD ligation and ischaemia–reperfusion groups) was the re-establishment of blood flow in the parent coronary vessel and thus elimination of the need for collateral flow from the microvasculature, it is reasonable to ascribe the predominant basis of the differences seen between the two groups studied to effects on the collateral circulation. In the unloaded permanently ligated hearts, the reduction of MV0_2_ due to mechanical unloading interferes with the autoregulatory stimulus necessary for the augmentation of collateral microcirculation thus perpetuating the ischaemic insult, leading to a rise in fibrosis. In the unloaded reperfused hearts, however, coronary flow from the collateral microcirculation is not required, and thus, the beneficial effects of unloading-induced reduction in MV0_2_ predominates with the amelioration of myocardial reperfusion injury and generation of a neurohumoral environment conducive for cardiomyocyte proliferation.

Whilst this hypothesis requires further validation, previous studies have also reported increase in myocardial fibrosis with mechanical unloading after MI, showing this to be associated with a reduction in microvascular luminal diameter and coronary flow reserve [39, 44].

To better understand the acute effects of mechanical unloading on the propagation of myocardial fibrosis after permanent coronary ligation and coronary reperfusion, the expression profile of αSMA in each group was determined. At day 7, mechanical unloading coupled with coronary reperfusion led to a statistically significant decrease in αSMA levels. This suggests that the activity of myofibroblasts might be downregulated when mechanical unloading is coupled with coronary reperfusion with consequent decrease in ECM protein deposition. This might also explain the amelioration of the increase in myocardial fibrosis that is seen in the permanently ligated group after unloading.

The findings from this study supports the notion that mechanical unloading promotes the reversal of the injured adult heart back to its foetal haemodynamic phenotype with a reduction in mechanical load and myocardial wall stress and a resultant increase in its hyperplastic (as opposed to hypertrophic) growth that favours cardiomyocyte proliferation [16, 39].

The synergistic effect of coronary reperfusion and mechanical unloading in reducing the propagation of myocardial fibrosis and stimulating significant proliferation of new cardiomyocytes has major clinical implications as it suggests that after acute MI, mechanical unloading at the time of primary PCI could potentiate the hearts own intrinsic capacity for self-repair. It also adds to the growing body of evidence supporting the use of short-term mechanical circulatory support for the management of AMI in high-risk patients undergoing PCI or surgery. Outcomes of current ongoing trials such as PROTECT IV RCT, RESTORE IV, and the IMPACT trial are much awaited [18, 58]

## Study limitations

The use of Ki67 as a proliferative marker in this study is limited by the fact that whilst it identifies cells at any of the phases of the cell cycle aside from G0 (G1, S, G2, and M phases), it does not determine the presence of true cell division as opposed to binucleation or multinucleation. For this reason, we only counted one nucleus per cell and only counted cardiomyocytes with well-defined borders using WGA staining. Thus, we have possibly underestimated the total number of possible proliferating cardiomyocytes in this study. Future studies using markers of mitosis such as Aurora B and or phosphorylated histone will be beneficial. Other limitations include the impact of anaesthesia, recovery, and surgical stress on data variability, and the loss of temporal appreciation of the dynamics of fibrosis and proliferation due to the single (7 day) time point chosen for analysis. Future studies will investigate the evolution of remodelling over several weeks/months.

Furthermore, the small sample size of 5 and findings regarding αSMA protein expression and pYAP which were based on analyses from only three animals per group, may limit the robustness and generalizability of these results.

Finally, this study was performed using an animal model of mechanical unloading that may differ from the mechanical unloading with LVAD in amount of unloading, its timing, lack of systemic effects, denervation, and others. [25] To extend our conclusions to the clinic, further studies in patients treated with LVADs are warranted.

## Conclusion

The findings described in this study have significant implications for the management of acute myocardial infarction as it is the first report to show that coupling mechanical unloading with coronary reperfusion not only provides haemodynamic support for these high-risk patients but also help stimulate the hearts intrinsic capacity for self-repair. Further studies are needed to corroborate these findings.

## Supplementary Information

Below is the link to the electronic supplementary material.

Supplementary file1 (DOCX 3430 KB)
