# Supplementary material for: Mechanical unloading coupled with coronary reperfusion stimulates cardiomyocyte proliferation and prevents unloading-induced fibrosis after myocardial infarction
Source: Basic Res Cardiol. 2025 Nov 22;121(1):43–57. doi: 10.1007/s00395-025-01147-4 (PMC12804340; doi:10.1007/s00395-025-01147-4)
Supplement: Supplementary file 1 — Supplementary file1 (DOCX 3430 KB) [file 395_2025_1147_MOESM1_ESM.docx]

Supplementary Material

**Mechanical unloading coupled with coronary reperfusion reduces fibrosis and** **stimulates cardiomyocyte proliferation after myocardial infarction**

Sean O. Bello, MD, PhD, FRCS-CTh; Charanjit Singh, PhD; Filippo Perbellini, PhD; Prakash P. Punjabi, MD, FRCS-CTh; Cesare M. Terracciano, MD, PhD.

Corresponding author: Professor Cesare Terracciano

Email: c.terracciano@imperial.ac.uk

Address: Imperial Centre for Translational and Experimental Medicine, Hammersmith Hospital, National Heart & Lung Institute, Imperial College London, W12 0NN

**METHODS**

MI and Coronary reperfusion model

Lewis rats are inbred and as such were chosen to avoid the need for immunosuppression after transplantation. They have also been shown to develop large infarct sizes, LV chamber dilatation, and progress to LV dysfunction with much lower mortalities more consistently following coronary artery ligation compared to several other strains of rat. ^1^ These animals have been extensively used for similar studies thus facilitating standardisation. ^2^ ^3^

The operative steps were implemented as previously described. ^4^ In brief, syngeneic male Lewis rats were anaesthetised using the inhalation anaesthetic agent isoflurane at 3-5% for induction & 1.5-2.5% for maintenance. The anaesthetic circuit used was the Bain’s co-axial facemask. Oxygen flow was kept at 3L/min for induction and 1.5-2L/min for maintenance. They were then intubated using a 16 G vascular cannula and general anaesthesia achieved via a volume controlled rodent ventilator (Model 683 Rodent ventilator, Harvard 101Apparatus Ltd, UK). The tidal volume was kept at about 6-8ml/kg with a ventilatory rate of about 40 breaths/min and body temperature of 37^0^ C. Intraoperative pain relief consisted of a single subcutaneous dose of vetergesic (buprenorphine) at 0.12mg/kg. Adequate hydration was achieved by giving 1ml bolus of warm saline subcutaneously every hour.

Once adequacy of anaesthesia was confirmed by loss of pedal reflexes, the animal was prepped and draped, a left anterolateral thoracotomy was made over the fourth rib, and the proximal left anterior descending artery (LAD) was ligated using 6/0 prolene sutures.

After coronary artery ligation, visual assessment of the apex of the heart was carried out to determine onset of the infarct. The lungs were inflated and the chest wall temporarily closed.

Animals were then left on the ventilator for 90 mins. This was taken as sufficient time to allow transmural myocardial infarction to occur.

In the permanently ligated group the animal was recovered after 90 mins. In the reperfusion (R) subgroup, the chest wall was reopened after 90 mins and the ligature to the proximal LAD released to return blood flow to the infarct territory. The chest wall was then closed and the animal recovered as previously described.

Quantification of extent of myocardial injury

Histological analysis of infarct size was obtained from each study sample as evidence of extent of myocardial injury.

Mechanical unloading model

The heterotopic abdominal heart lung transplantation technique was used as the model of mechanical unloading.

It involves anastomosing the heart and lung en bloc from a donor onto the abdominal aorta of a healthy recipient. ^5^ Only a single anastomosis of the ascending aorta of the donor heart to the abdominal aorta of the recipient is completed. As the inferior vena cava (IVC) and superior vena cava (SVC) are ligated and the pulmonary circulation remains intact, the LV receives additional blood from the right ventricle (RV), via the pulmonary veins after passing through the lungs as illustrated in figure S1.

Figure S1: Schematic representation of HAHLT. In this preparation the donor heart and lung en bloc is transplanted by anastomosing the ascending aorta of the donor to the abdominal aorta of the recipient. The LV is only partially unloaded here because the venous return from the coronary sinus travels through the lungs and empties into the left atrium and eventually the LV. Figure reproduced with permission from Ibrahim et al 2013. ^6^

The operative steps are as previously described. ^6^ In brief, following preparation and establishment of anaesthesia, the torso was prepped with povidone iodine and draped. (Fig. S2) Intraoperative pain relief consisted of a single subcutaneous dose of vetergesic (buprenorphine) at 0.12mg/kg. Adequate hydration was achieved by giving 1ml bolus of warm saline subcutaneously every hour.


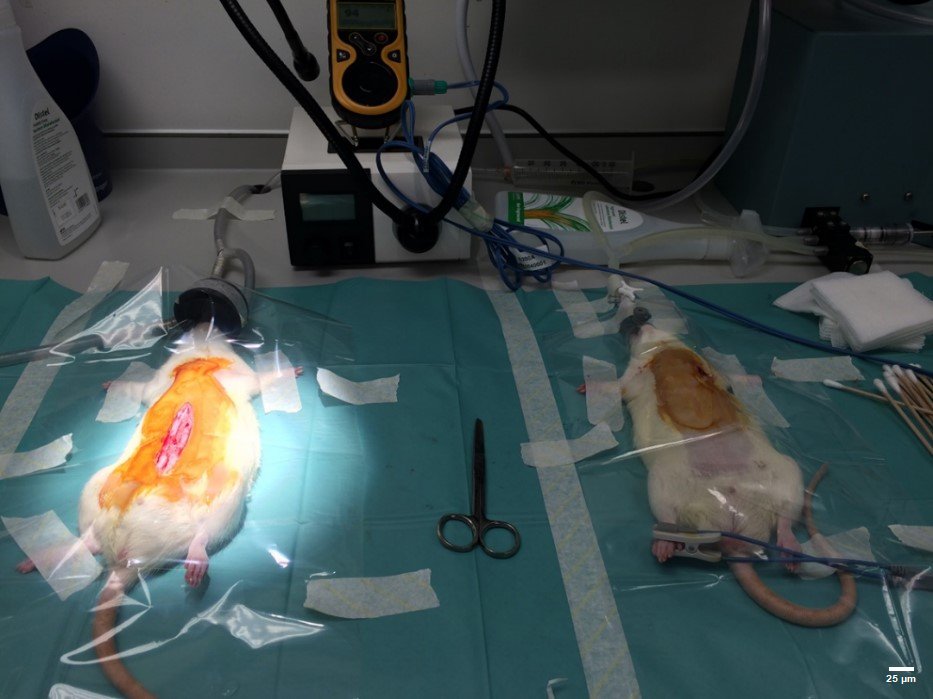


Figure S2: Two Lewis rats being prepared for HAHLT. The rat on the right has had 90 mins of LAD ligation via a left mini thoracotomy (white arrow). The rat on the left was the recipient with a midline laparotomy (black arrow) being initiated.

The recipients’ abdominal aorta was accessed via a midline laparotomy incision and with the aid of a microscope, a 0.5cm midline longitudinal aortotomy was carried out and prepared for anastomosis. The donor abdominal aorta was then accessed and adequate heparinisation was achieved by injecting heparin at 10000 IU/Kg into the IVC using a 30G needle. The donor abdominal aorta was then cannulated with a 20-gauge arterial line (Leadercath arterial, Vygon UK Ltd, FSQ049) using a catheter-over-wire technique and this was used to inject 50mls of cold St Thomas II cardioplegia solution into the donor circulation thus arresting the donor heart (permanently ligated or R) in diastole. The IVC was simultaneously transected to prevent overload and euthanasia was achieved via exanguination. The sternum was cut open and the heart explanted after ligating the IVC and SVC using 4/0 Mersilk sutures. Figures S3 & S4 show the permanently ligated heart and the reperfused hearts at the point of harvest for transplantation.

Note that after infusing crystalloid cardioplegia solution, the infarct territory (B in figure S3) can be seen clearly in the permanently ligated heart, confirming obstruction to flow through the ligated vessel. In the reperfusion group this area is blanched by the crystalloid cardioplegia solution (B in figure S4) confirming return of flow through the previously ligated coronary artery.


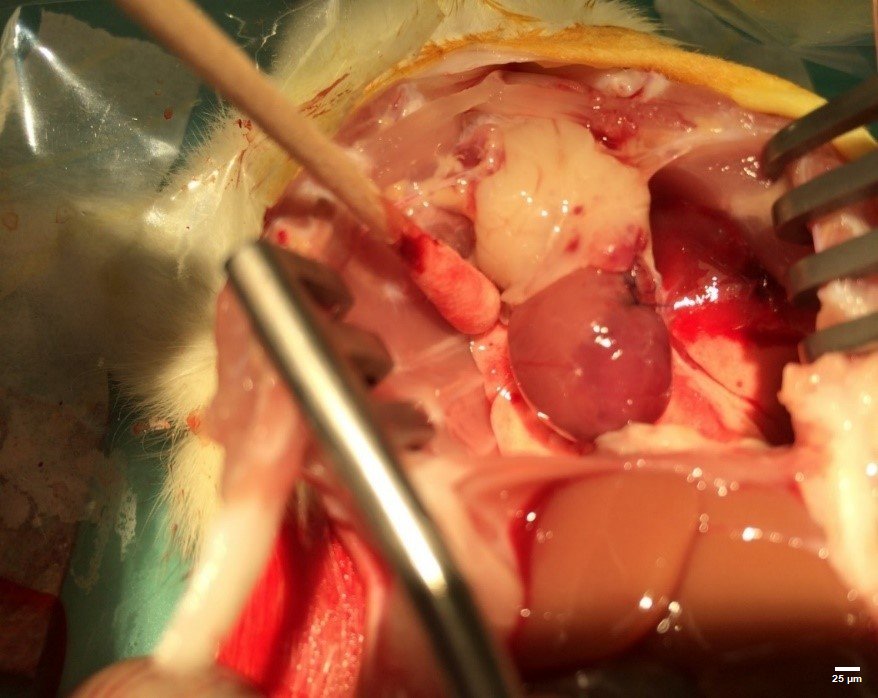


A

B

Figure S3: Thoracic cavity of the rat opened revealing the permanently ligated heart after infusion of St Thomas II cardioplegia solution showing blockage of flow to the infarct region (B). The arrow is pointing at the LAD ligature. A swab stick is used to expose areas of the heart blanched by the infused cardioplegia (A) thus providing a clear indication of the region with an occluded coronary circulation (B).


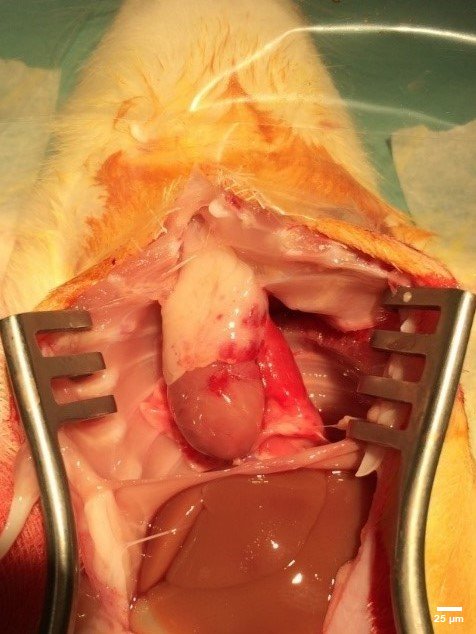


B

A

Figure S4: Ischaemia/Reperfusion heart after infusion of St Thomas II cardioplegia solution showing return of flow to the infarct region. There is now no clear demarcation between regions (A) and (B) indicating return of coronary flow through the LAD after removing the ligature (arrow).

The donor aorta was cut into shape with microscissors and anastomosed to the recipient’s abdominal aorta using 8/0 prolene sutures. The bulldog clamps were removed at completion and haemostasis achieved. The donor heart beat returned after a few seconds. The operation was concluded by closing the abdominal wall in layers.

Heart harvest and preparation

At day 7, animals were weighed and adequately anaesthetised as described above. For the loaded infarcted heart harvest, the animal was sacrificed using schedule 1 cervical dislocation. The chest wall was cut open rapidly and the heart explanted and inserted into cold heparinised St Thomas II cardioplegia solution. For the unloaded heart harvest, the recipient’s abdominal aorta was clamped above and below the point of anastomosis to the donor aorta using bulldog clamps. The unloaded heart was then removed and rapidly inserted into cold St Thomas II cardioplegia solution. The bulldog clamps were then released and euthanasia achieved via exanguination. The recipient’s sternum was then cut open and its heart removed and also rapidly arrested in cold cardioplegia solution to be used as control.

The lungs and atria were excised after heart explantation and the ventricle weighed. The ventricular weight (VW) to body weight (BW) ratio (VW:BW) for each heart was recorded.

The ventricle was then placed in an acrylic zivic rat heart slicer matrix with 2mm coronal section slice intervals (Zivic instruments, HSRA002-1) and sliced into 5 coronal segments A-E. (Fig. S5)

Figure S5: Zivic slicer (thick arrow) provides 2mm slices of heart tissue A-E (thin arrow). Right sided image reproduced with permission from Ellison et al 2011. ^7^

Whilst still submerged in cold St Thomas II cardioplegia solution, the left ventricular aspect of segments C & E were each further divided into their respective chambers: anterior, posterior, and septal to facilitate regional assessment of protein expression. The tissues were then snap frozen in liquid nitrogen and stored in -80^0^C for protein analysis. Segments A, B & D were frozen in the Optimal Cutting Temperature compound (OCT) and also stored in -80^0^C for histological analysis.

Experimental groups

In this study, the recipient’s healthy heart served as control (CTR). Animal models of acute MI and HAHLT were divided into 4 broad groups:

1. Acute MI Loaded (AMI-L) – here permanently ligated hearts were explanted for analysis 7 days after coronary ligation.
2. Acute MI Reperfusion Loaded (AMI/R-L) – here reperfused hearts were explanted for analysis 7 days after coronary ligation.
3. Acute MI Unloaded (AMI-U) – here permanently ligated hearts were explanted 90 minutes after coronary ligation and immediately mechanically unloaded via HAHLT. These hearts were then explanted for analysis 7 days after transplantation.
4. Acute MI Reperfusion Unloaded (AMI/R-U) – here reperfused hearts were explanted 90 minutes after coronary ligation and immediately mechanically unloaded via HAHLT. These hearts were then explanted for analysis 7 days after transplantation.

All animals were randomly assigned codes and these were blinded to the primary investigator who carried out all the experiments.

Frozen tissue & Cryosections

Each heart segment was cleaned in cardioplegia solution and submerged flat in the base of a 5ml sterilin tube (ThermoFisher scientific, Z5PS) containing 1ml of OCT compound. The tissue was then snap frozen in isopentane mixed with dry ice and stored in -80^0^C ready for sectioning.

15μm cryosections were obtained using a cryostat at proper operating temperature of -20^0^C to -30^0^C. These were then mounted onto glass slides (SuperFrost Plus, Thermo Scientific, 630-0954), 3 sections per slide and stored in -80^0^C ready for immunohistochemistry.

Histological analysis of frozen tissue sections for the quantification of myocardial fibrosis

The 15μm cryosections prepared as described above were processed with histological stains to assess the degree of fibrosis in the rat models of MI. Sirius red/Fast green collagen staining was employed using a standardised protocol. In brief, the glass slides containing tissue sections are taken from -80^0^C storage and hydrated in ethanol gradations i.e. 100% ethanol, 70% ethanol, and distilled water each for 20s duration. The Sirius red/Fast green solution was filtered to remove debris and the slides were submerged in solution for 35 mins, agitating 2-3x every 10 mins. The stained slides were then washed in 100% ethanol and again agitated 2x every 20s. Under the fume cupboard, the slides were immersed in two separate xylene containing troughs, each immersion lasting about 5 mins to 10 mins to eliminate glass-like appearance on the slides. Using a DPX mountant (Sigma-Aldrich, 06522-100ML), coverslips were applied and slides were allowed to dry overnight at room temperature in preparation for imaging.

Widefield microscopy and thresholding on ImageJ software for the quantification of myocardial fibrosis

Images of the stained sections were obtained with the widefield microscope as illustrated in figure S6. With the aid of imageJ software, infarct size (IS) and area at risk (AAR) were determined using planimetry. The IS was the area stained red (Fig. S6A) whilst the AAR was the whole left ventricle (Fig. S6B).


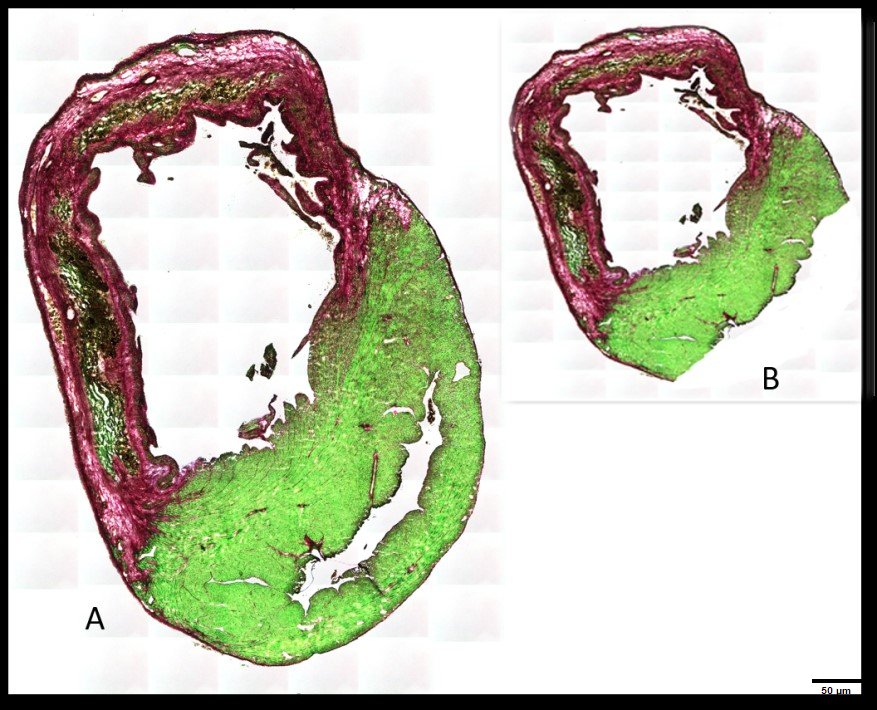


Figure S6: Sirius red/Fast green histological stain of a cross-section of the infarcted rat heart showing the extent of necrotic cell death and collagen deposition (red stain) and areas of viable myocardium (green stain). Image B (LV free wall and septum) illustrates the area at risk of infarction following proximal LAD ligation. Left ventricle (thin arrow), Right ventricle (thick arrow). Total fibrosis was obtained from the whole left ventricle (B)

The thresholding tool on imageJ was used to accentuate the contrast between the areas of collagen deposition and healthy myocardium in the left ventricle (Fig. S6B) and hence quantify the extent of myocardial fibrosis.

Mode of detection of proliferating cells

Immunohistochemistry staining of frozen tissue sections using the antibody Ki67 was the technique employed in this study to determine cell proliferation. Ki67 is a protein known to be strictly associated with cell proliferation and has been used as the marker of choice in several studies as it is exclusively expressed during the active phases of the cell cycle i.e. G1, S, G2, and mitosis, but is absent in the resting phase (G0) and in differentiated cells. Ki67 has been shown to be a more sensitive and specific marker of cell proliferation compared to other markers such as the Proliferating Cell Nuclear Antigen (PCNA) or the thymidine analogue Bromodeoxyuridine (BrdU). The expression of the latter two have been shown to be significantly affected by DNA damage and repair.

Ki67 immunohistochemistry is however limited in that it only provides information about the proliferative rate of cells at the point of heart harvest. As such it does not provide an accurate representation of the full rate at which cells are proliferating. It is thus important to note that the data provided here represents an underestimation of the actual proliferative capacity of the adult heart.

Immunohistochemistry was the preferred approach as opposed to flow cytometry as immunohistochemistry preparations afford regional assessment of proliferation, and deals with the confounding issue of multinucleation as tissues are cut in transverse section meaning only one nucleus or none was visible. As the study was carried out on frozen tissue cross sections, only one Ki67 positive nucleus per cell was counted.

Immunohistochemistry of frozen tissue sections for the analysis of cardiomyocyte proliferation

The immunohistochemistry staining technique was applied on frozen tissue sections to facilitate regional assessment of cardiomyocyte proliferation. The cellular marker Ki67 was used in addition to the cardiomyocyte marker cTnT, the stromal cell marker vimentin, and the membrane marker WGA. The procedure was completed using a standardised protocol. In brief, glass slides containing 15μm tissue sections were submerged into pre-cooled methanol (-20^0^C) for 5 mins. Afterwards, 1ml of 1.5% triton in 10mM phosphate buffered saline (PBS) was spread over each glass slide to permeabilise the tissue for 20 mins. During this time, blocking solution made up of 1% bovine serum albumin (BSA) and 0.1% triton both in PBS was freshly prepared. After 20 mins of permeabilization the tissue section was submerged in blocking solution, covered in foil and kept in the fridge at 4^0^C for 1 hour. The slides were then drained of fluid and a circular hydrophobic barrier was drawn around the tissue using a PAP pen (Sigma-Aldrich, Z377821-1EA). The tissue sections were kept overnight at 4^0^C after introducing the following primary antibodies: rabbit anti-Ki67 (1:300, Abcam, ab16667), mouse anti-cardiac troponin (1:300, ThermoFisher, MA512960), and chicken anti-vimentin (1:3000, ThermoScientific, PA1-10003).

After a minimum of 12 hours the tissues were washed with PBS 2x for 15 mins each and the following secondary antibodies from ThermoFisher were introduced at a dilution of 1:1000: Alexa 488 donkey anti-rabbit (A21206), Alexa 546 donkey anti-mouse (A10036), Alexa 568 goat anti-chicken (A11041) and Alexa Fluor 555 conjugate (wheat germ agglutinin) (1:100 ThermoFisher, W32464). The sections were kept at 4^0^C covered in foil for 1 hour. This was followed by 3 washes in PBS for 15 mins each. Finally, the tissue sections were covered with the nuclei stain Hoechst 33342 (1:1000, Thermoscientific, H3570) for 10 mins at 4^0^C, washed 2x with PBS for 15 mins each, and with the aid of 50% glycerol in PBS as mounting media, borosilicate rectangular glass coverslips (22mm x 40mm, VWR, 631-0138) were placed over the tissue and secured around the edges with nail varnish. Prepared samples were then kept at 4^0^C ready for imaging.

Confocal & Widefield microscopy

Following immunochemical staining with cTnT, Ki67, WGA, vimentin, and Hoechst as described above, samples were imaged under confocal microscopy with z-stacking tool utilised to verify cardiomyocyte nuclei. Widefield microscopy was then used to aid regional analysis of cell proliferation. Proliferating cells were counted manually from three distinct regions of the stained left ventricle and these include: Anterior infarct borderzone (between the anterior wall of the right ventricle and septum, and the anterior aspect of the left ventricular free wall), Posterior infarct borderzone (between the posterior wall of the right ventricle and septum, and the posterior aspect of the left ventricular free wall), and Distant myocardium (midpoint of the interventricular septum). Each region counted was about 1.4mm^2^ and contained approximately 2000 cardiomyocytes.

Strict criteria were followed to avoid counting non-cardiomyocyte nuclei or counting more than one nucleus per cell. These criteria were as follows:

1. Thresholding tool on imageJ analysis software was used to eliminate autofluorescence.
2. Only regions with cardiomyocytes cut in transverse section were included in the count and only one positive nucleus per cell was counted.
3. Only cardiomyocytes with positive nuclei positioned centrally in the cell or well clear of the cell membrane (stained with WGA) were counted.

Western blotting

Western blotting was used to determine the dynamic expression profiles of proteins involved in the regulation of myocardial fibrosis and cell proliferation such as αSMA and pYAP respectively.

**RESULTS**

The increase in myocardial fibrosis after acute myocardial infarction and mechanical unloading that is observed in the permanently ligated hearts as opposed to the ischaemia-reperfused hearts can be seen in figure S7(A-D).


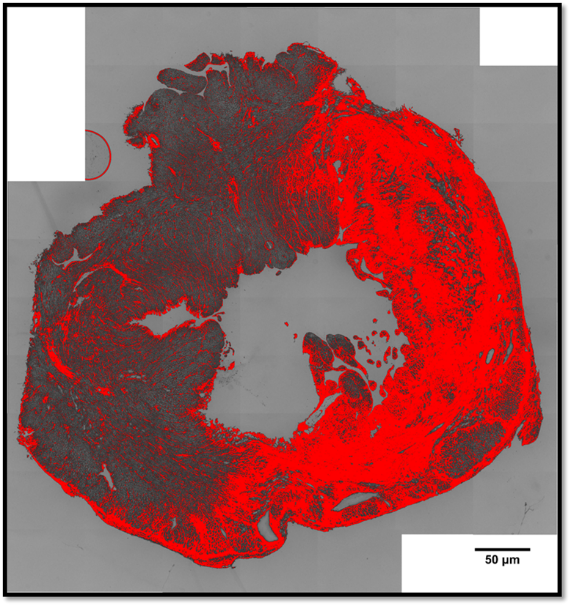

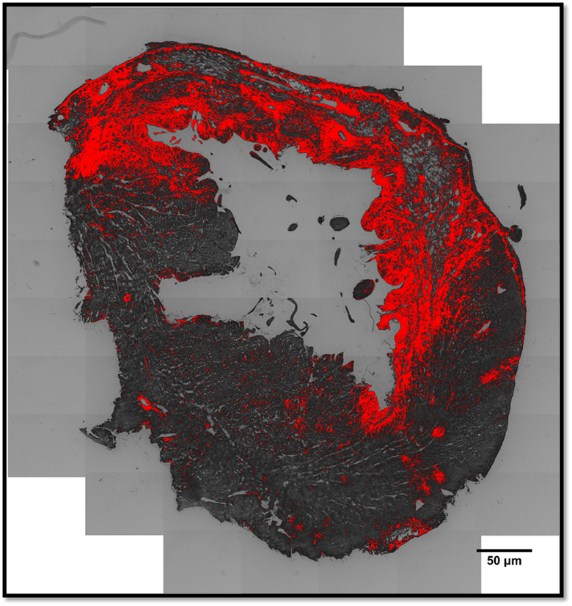


B

A


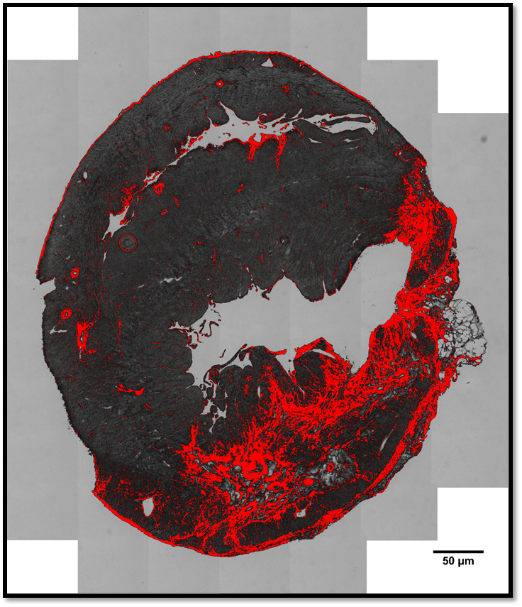

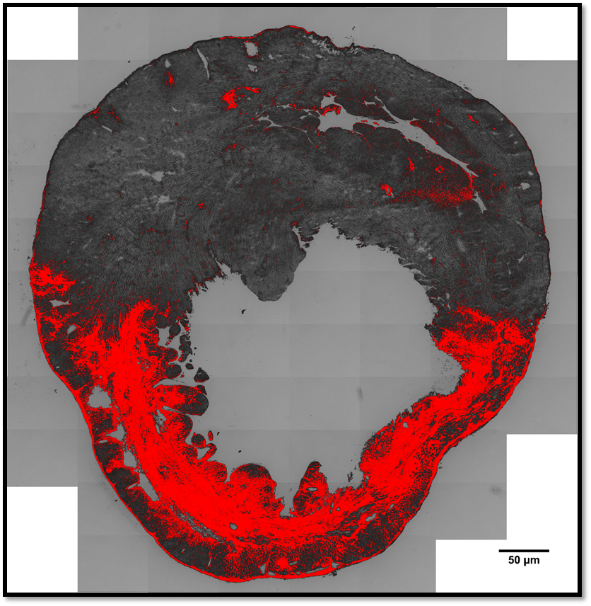


D

C

Figure S7: (A) Myocardial fibrosis in an AMI-L sample at day 7 with increase in fibrosis evident after mechanical unloading in the AMI-U sample at day 7 (B). In the ischaemia-reperfusion group however, mechanical unloading in the AMI/R-U (D) did not significantly change the extent of fibrosis compared to that in the loaded AMI/R-L heart (C) at day 7. Areas of fibrosis was determined by Sirius red staining. In the unloaded reperfused hearts areas furthest from the infarct site showed less fibrosis (D) compared to that observed in the unloaded permanently ligated hearts (B).

**REFERENCES**

1. Liu Y, Yang X, Nass O, Sabbah H a NN. Chronic heart failure induced ligation in Lewis inbred rats by coronary artery. *Am J Physiol*. 1997;272(2Pt 2):H722-7.

2. Ibrahim M, Navaratnarajah M, Siedlecka U, et al. Mechanical unloading reverses transverse tubule remodelling and normalizes local Ca 2+-induced Ca 2+release in a rodent model of heart failure. *Eur J Heart Fail*. 2012;14:571-580. doi:10.1093/eurjhf/hfs038

3. Navaratnarajah M, Ibrahim M, Siedlecka U, et al. Influence of ivabradine on reverse remodelling during mechanical unloading. *Cardiovasc Res*. 2013;97:230-239. doi:10.1093/cvr/cvs318

4. Gross L, Beck C. Experimental myocardial infarction. I. A method of coronary occlusion in small animals. *Ann Surg*. 1954;140(5):675-682.

5. Fu X, Segiser A, Carrel TP, Tevaearai Stahel HT, Most H. Rat Heterotopic Heart Transplantation Model to Investigate Unloading-Induced Myocardial Remodeling. *Front Cardiovasc Med*. 2016;3(October). doi:10.3389/fcvm.2016.00034

6. Ibrahim M, Navaratnarajah M, Kukadia P, et al. Heterotopic abdominal heart transplantation in rats for functional studies of ventricular unloading. *J Surg Res*. 2013;179(1):e31-e39. doi:10.1016/j.jss.2012.01.053

7. Ellison GM, Torella D, Dellegrottaglie S, et al. Endogenous cardiac stem cell activation by insulin-like growth factor-1/hepatocyte growth factor intracoronary injection fosters survival and regeneration of the infarcted pig heart. *J Am Coll Cardiol*. 2011;58(9):977-986. doi:10.1016/j.jacc.2011.05.013
